# Supplementary figures and images for: Structural and Functional Analysis of the MADS-Box Genes Reveals Their Functions in Cold Stress Responses and Flower Development in Tea Plant (Camellia sinensis)
Source: Plants (Basel). 2023 Aug 13;12(16):2929. doi: 10.3390/plants12162929 (PMC10458798; doi:10.3390/plants12162929)

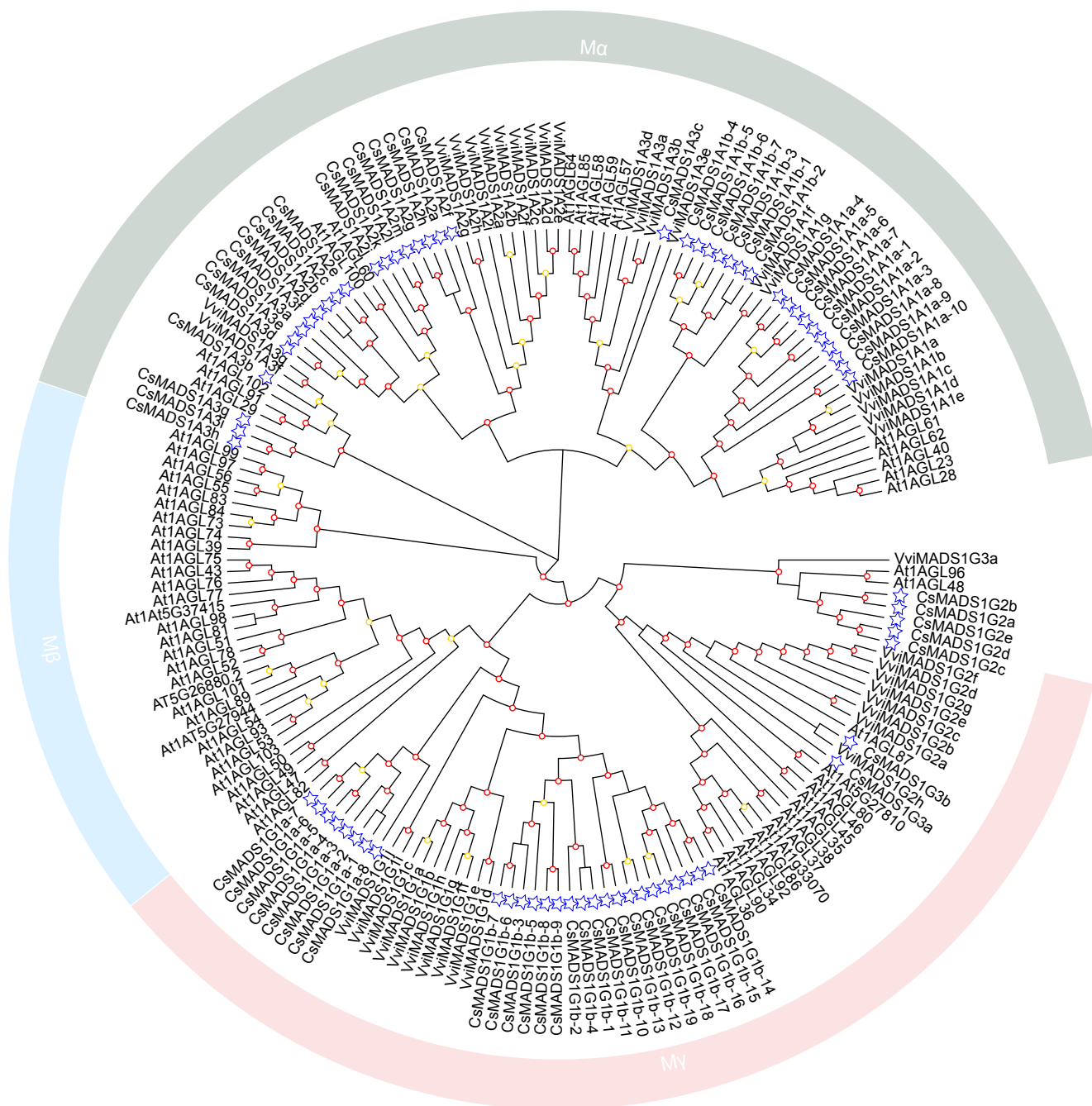

Supplement: Supplementary file 1 [file plants-12-02929-s001.zip › plants-2499603-supplementary/Supplimenatry Figure S2.pdf]

## Detailed Identification pipeline of tea MADS-BOX gene family

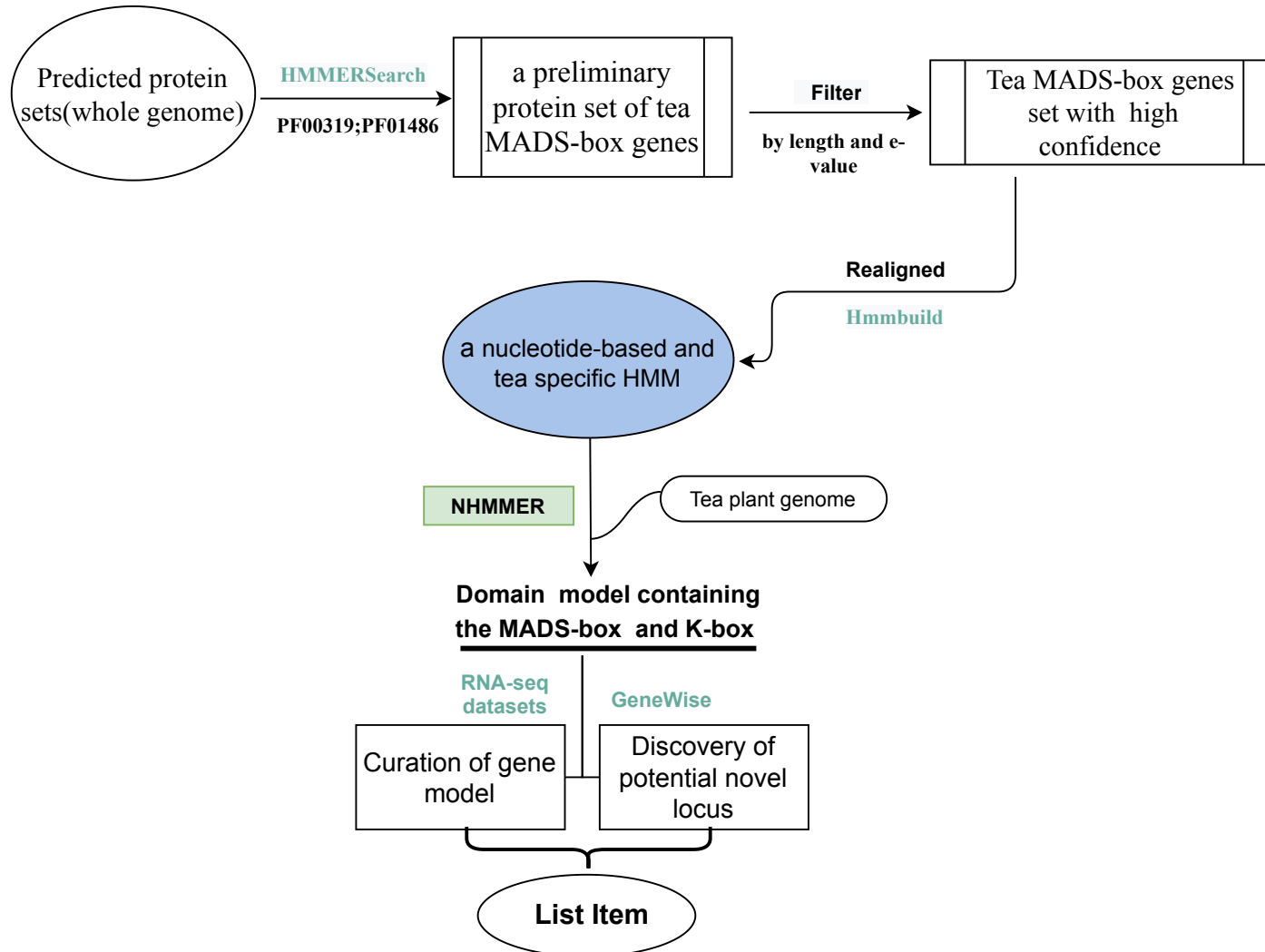

Supplement: Supplementary file 1 [file plants-12-02929-s001.zip › plants-2499603-supplementary/Supplimentary Figure S1.pdf]
